# Amino-functionalized (meth)acryl polymers by use of a solvent-polarity sensitive protecting group (Br-*t*-BOC)

**DOI:** 10.3762/bjoc.12.26

**Published:** 2016-02-10

**Authors:** Helmut Ritter, Monir Tabatabai, Markus Herrmann

**Affiliations:** 1 Institute of Organic Chemistry and Macromolecular Chemistry, Heinrich-Heine-University Duesseldorf, Universitaetsstraße 1, D-40225 Duesseldorf, Germany

**Keywords:** amino group protection, bromo-*tert*-butyloxycarbonyl, deprotection, free radical polymerization, (meth)acryl polymers, neighboring group effects, solvent polarity

## Abstract

We describe the synthesis of bromo-*tert*-butyloxycarbonyl (Br-*t*-BOC)-amino-protected monomers 2-((1-bromo-2-methylpropan-2-yl)oxycarbonylamino)ethyl (meth)acrylate **3a**,**b**. For this purpose, 2-isocyanatoethyl (meth)acrylate **1a**,**b** was reacted with 1-bromo-2-methylpropan-2-ol (**2a**). The free radical polymerization of (Br-*t*-BOC)-aminoethyl (meth)acrylates **3a**,**b** yielded poly((Br-*t*-BOC)-aminoethyl (meth)acrylate) **6a**,**b** bearing protected amino side groups. The subsequent solvolysis of the Br-*t*-BOC function led to the new polymers poly(2-aminoethyl (meth)acrylate) **8a**,**b** with protonated free amino groups. The monomers and the resulting polymers were thoroughly characterized by ^1^H NMR, IR, GPC and DSC methods. The kinetics of the deprotection step was followed by ^1^H NMR spectroscopy. The solvent polarity and neighboring group effects on the kinetics of deprotection are discussed.

## Introduction

**Figure 1:**
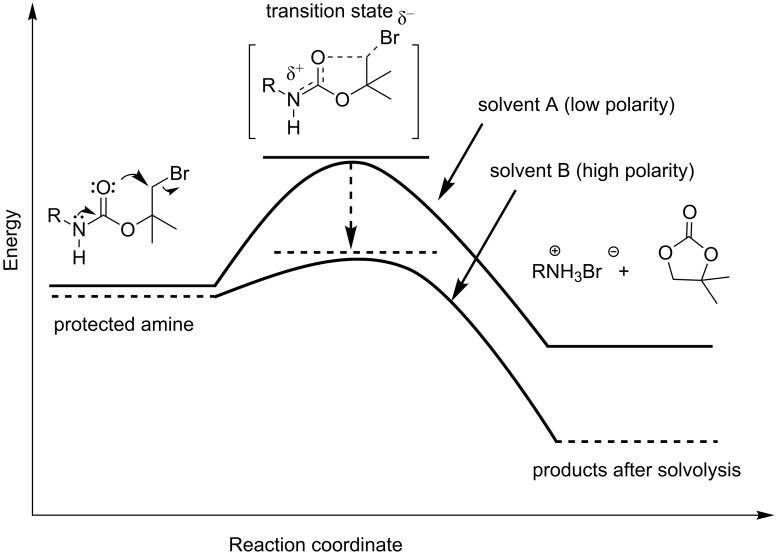
Schematic representation of a deprotection taking the relatively high polarity of the transition state and the solvent polarity into account: high activation energy in nonpolar solvents and low activation energy in polar solvents.

Amino groups are important functionalities in polymer chemistry, e.g., for hardening various epoxy resins [1]. However, they easily react in an undesired side reaction with electron-poor double bonds of (meth)acrylates [2]. Therefore, for the synthesis of amino-containing (meth)acrylic monomers and polymers suitable amino-protecting groups are required. Classical protecting groups such as ammonium salts, F-MOC, Z- or *t*-BOC, respectively are readily available. Regarding to this aspect, we published some papers about polymer protecting groups about three decades ago [3–7]. However, they are limited in application by certain restrictions on the deprotection conditions. Therefore, there is a continued interest in developing new protecting groups which can be cleaved by different mechanisms. Keeping this in mind, the bromo-*tert*-butyloxycarbonyl (Br-*t*-BOC) group represents the first known solvent-polarity sensitive amino-protecting group. As shown in Figure 1, this group is stable in nonpolar solvents because of high activation energy and easily decomposes in a more polar environment because of reduced activation energy. This effect is a result of an increased polarity of the transition state in comparison to the starting molecule (Figure 1). In contrast to the classical *t*-BOC protecting group, the Br-*t*-BOC group can be easily removed without pH adjustments. Since the published papers from L. A. Carpino [8–10] and a first practical application in peptide synthesis [11] this protecting group was quasi forgotten.

In the present work we report new Br-*t*-Boc-protected (meth)acrylic monomers and their polymerization through free radical polymerization. The kinetics of Br-*t*-BOC solvolysis of the monomers and polymers in a polar solvent are described.

## Results and Discussion

**Scheme 1:**
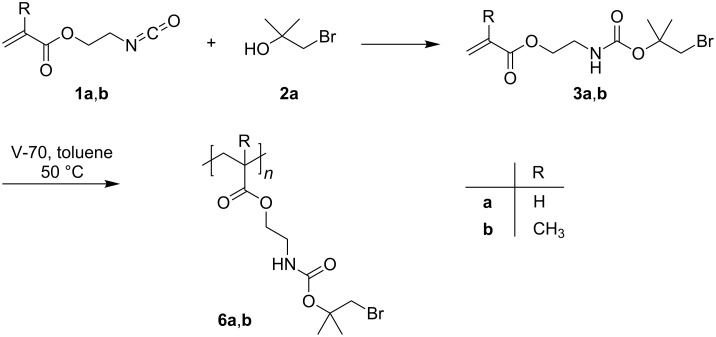
Synthesis of Br-*t*-Boc-protected monomers **3a**,**b** and homopolymers **6a**,**b**.

**Figure 2:**
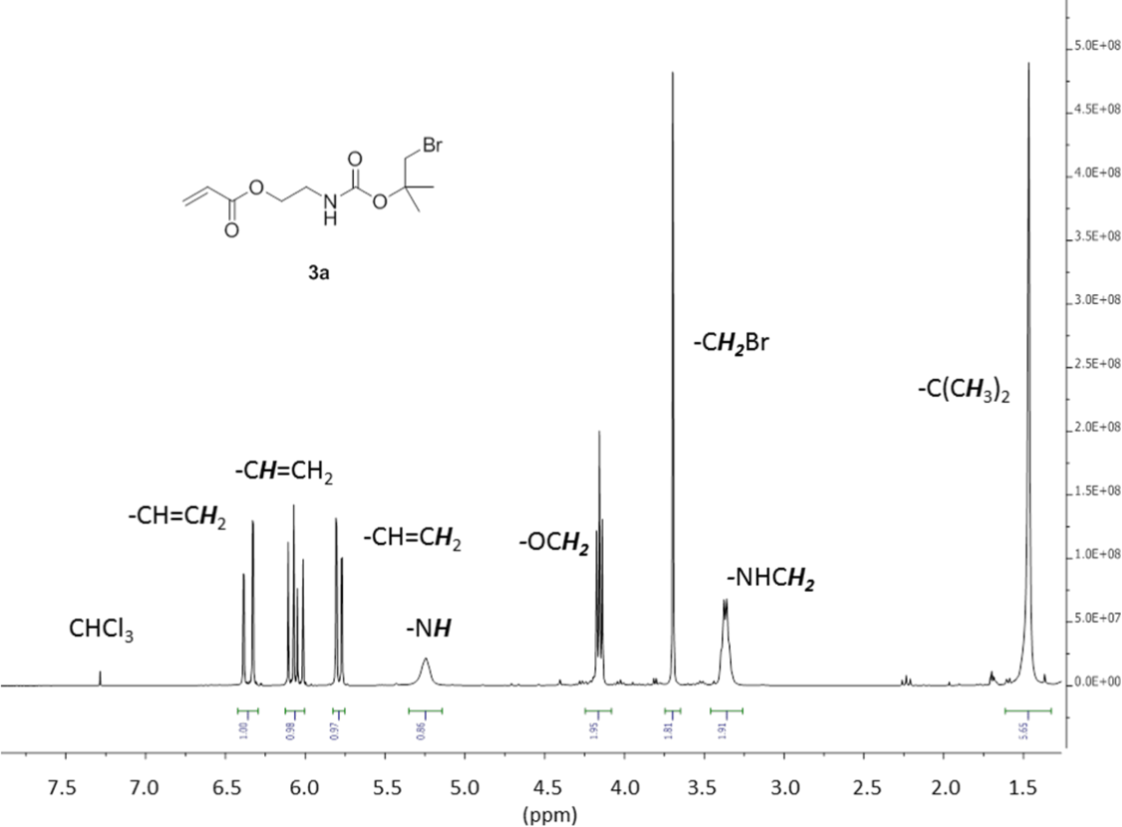
300 MHz ^1^H NMR spectrum of **3a** in CDCl_3_.

**Figure 3:**
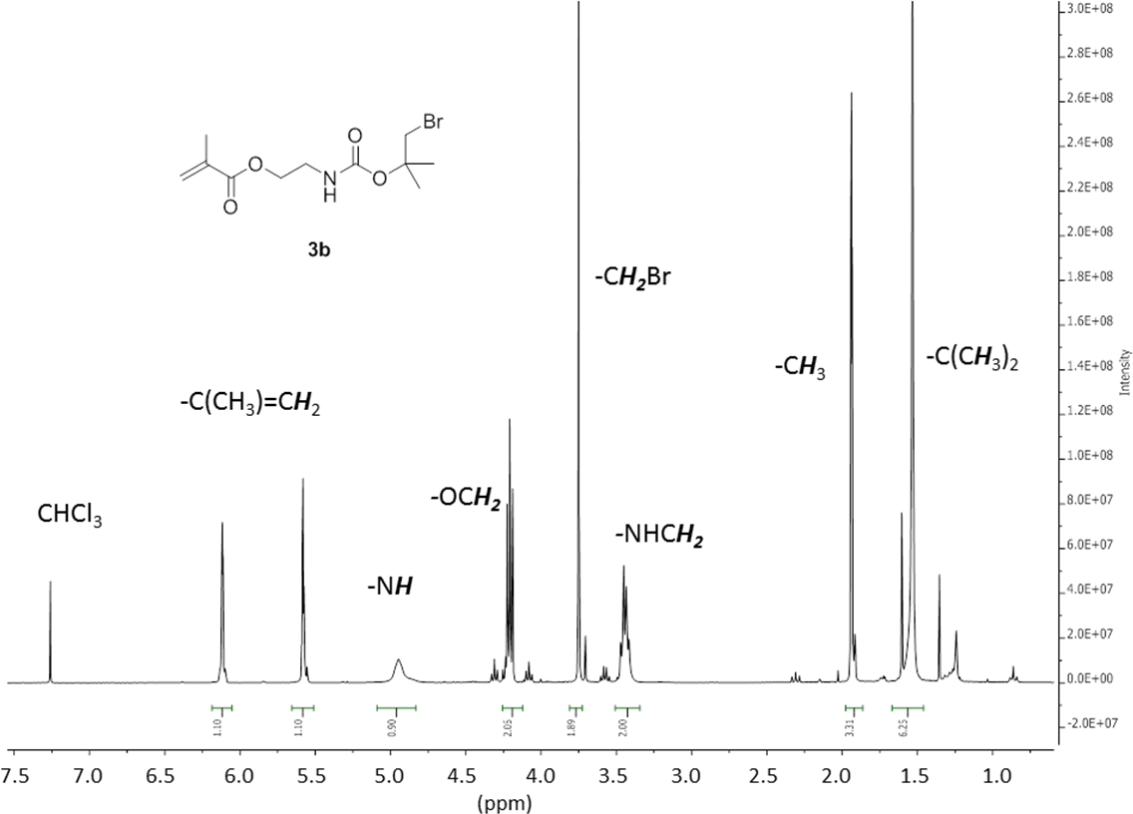
300 MHz ^1^H NMR spectrum of **3b** in CDCl_3_.

The Br-*t*-Boc-protected monomers 2-((1-bromo-2-methylpropan-2-yl)oxycarbonylamino)ethyl acrylate (**3a**) and 2-((1-bromo-2-methylpropan-2-yl)oxycarbonylamino)ethyl methacrylate (**3b**) were synthesized by the reaction of 1-bromo-2-methylpropan-2-ol (**2a**) with 2-isocyanatoethyl (meth)acrylate (**1a**,**b**) (Scheme 1). The success of the reaction can be easily shown by, e.g., the disappearance of the N=C=O peak of **1a** at 2260 cm^−1^ and the appearance of an N–H peak at 3350 cm^−1^ in the IR spectra. Pure samples of **3a**,**b** could be isolated by column chromatography and the ^1^H NMR spectra of **3a** and **3b** are shown in Figure 2 and Figure 3.

**Scheme 2:**
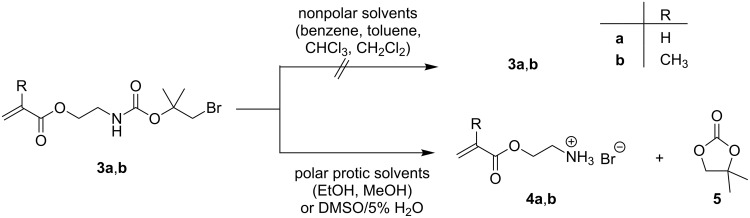
Stability of **3** in different solvents.

After dissolving monomers **3a** and **3b** in a polar solvent, they show the above mentioned self-cleaving process yielding the corresponding amine hydrobromide (**4a**,**b**) and the cyclic carbonate 4,4-dimethyl-1,3-dioxolan-2-one (**5**). As explained above, the protecting group undergoes a polar solvent driven intramolecular nucleophilic displacement (Figure 1). In contrast, in nonpolar solvents such as benzene, toluene, chloroform (CHCl_3_) or dichloromethane (CH_2_Cl_2_) **3a**,**b** are very stable, whereas in polar protic solvents such as ethanol (EtOH) or methanol (MeOH) the cleaving takes place very fast (Scheme 2). This deprotecting process can be directly followed using ^1^H NMR spectroscopy.

**Scheme 3:**
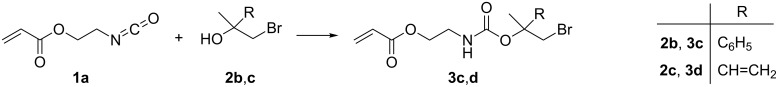
Synthesized derivative monomers **3c**,**d**.

Carpino assumed that, changing one or both of the methyl groups to bulkier substituents would significantly increase the cleaving process [10]. Thus, we attempted to replace one methyl group with a phenyl substituent simply by the reaction of **1a** with 1-bromo-2-phenylpropan-2-ol (**2b**). Although spectroscopic examinations gave evidence for the successful formation of the expected carbamate **3c**, only the corresponding amine hydrobromide **4a** was isolated as main product. This means that the amino-protecting group is extremely sensitive to decomposition. A third type of monomer was synthesized by reaction of **1a** with 1-bromo-2-methylbut-3-en-2-ol (**2c**) yielding 2-((1-bromo-2-methylbut-3-en-2-yl)oxycarbonylamino)ethyl acrylate (**3d**, Scheme 3). Although monomer **3d** is stable in nonpolar solvents, the cleaving process could not be followed by ^1^H NMR spectroscopy because of rapid decomposition in a polar solvent.

**Scheme 4:**
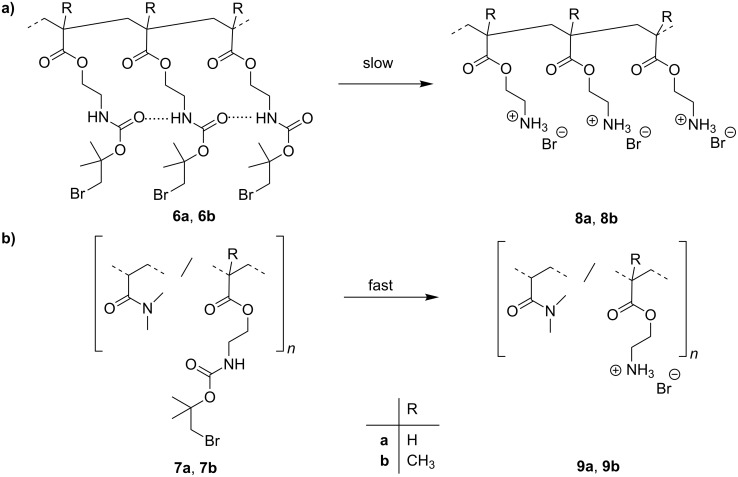
a) Self deactivation of homopolymer **6a**,**b** due to competing hydrogen interactions in comparison to b) copolymer **7a**,**b**.

Next, classical free radical polymerizations of **3a**,**b** were carried out in toluene as solvent at 50 °C using 2,2'-azobis(4-methoxy-2,4-dimethylvaleronitrile) (V-70) as initiator yielding homopolymers **6a**,**b**. This initiator is known to have a low decomposition temperature of 30 °C in toluene. Additionally, copolymers of **3a** and **3b**, respectively with *N*,*N*-dimethylacrylamide (**7**) were synthesized yielding **7a**,**b**. It was assumed that, since the cleaving process is coupled with cyclization to create the cyclic carbonate **5**, the homopolymer could be self-deactivated due to competing hydrogen-bond interactions of the urethane carbonyl group with neighboring groups. Since the comonomer should act as diluting agent a better access of the carbonyl group to the bromo-substituted carbon atom to fulfill the nucleophilic intramolecular displacement could be expected (Scheme 4).

**Figure 4:**
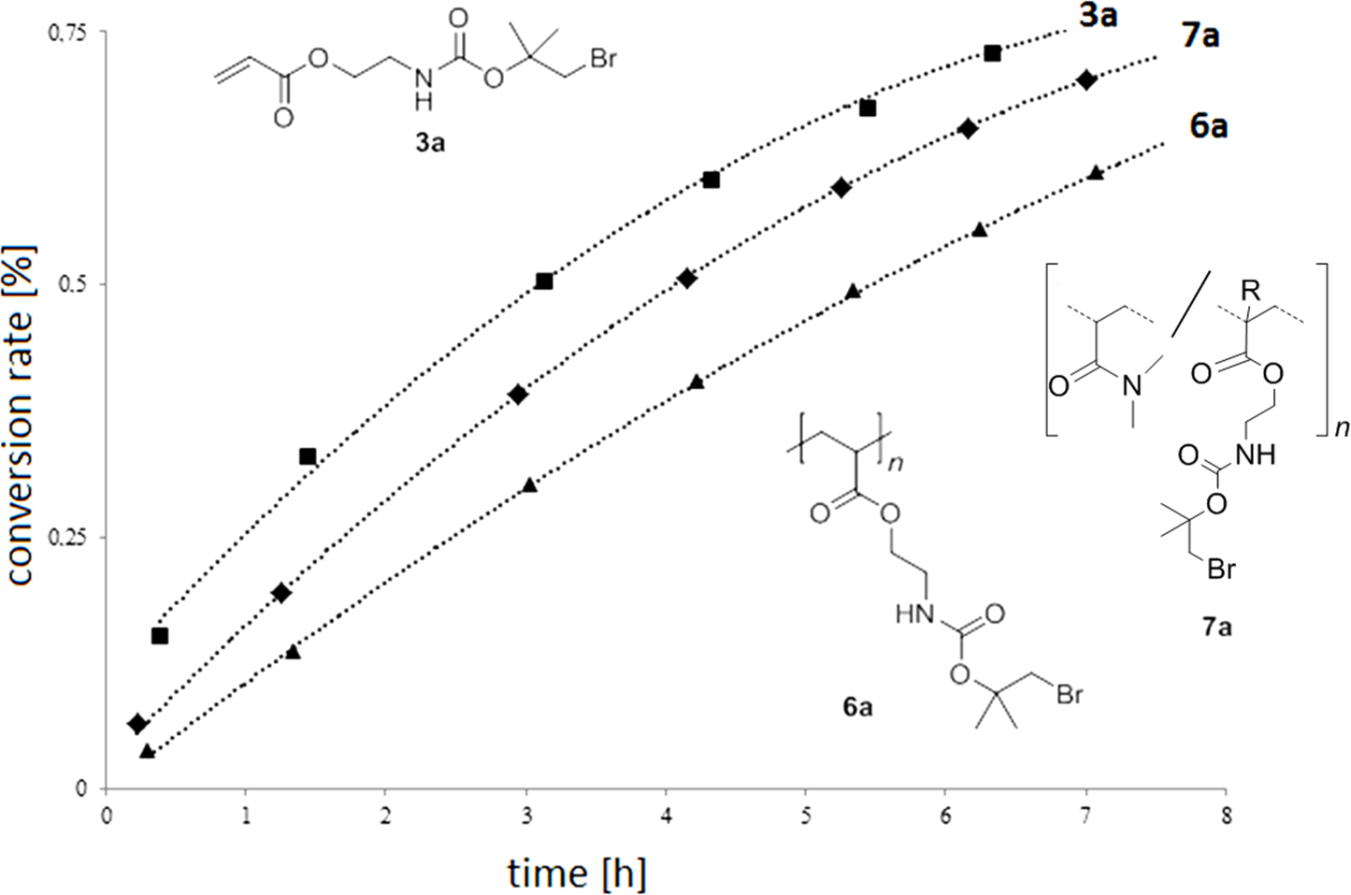
Kinetic studies of the deprotection of **3a**, **6a** and **7a**.

**Figure 5:**
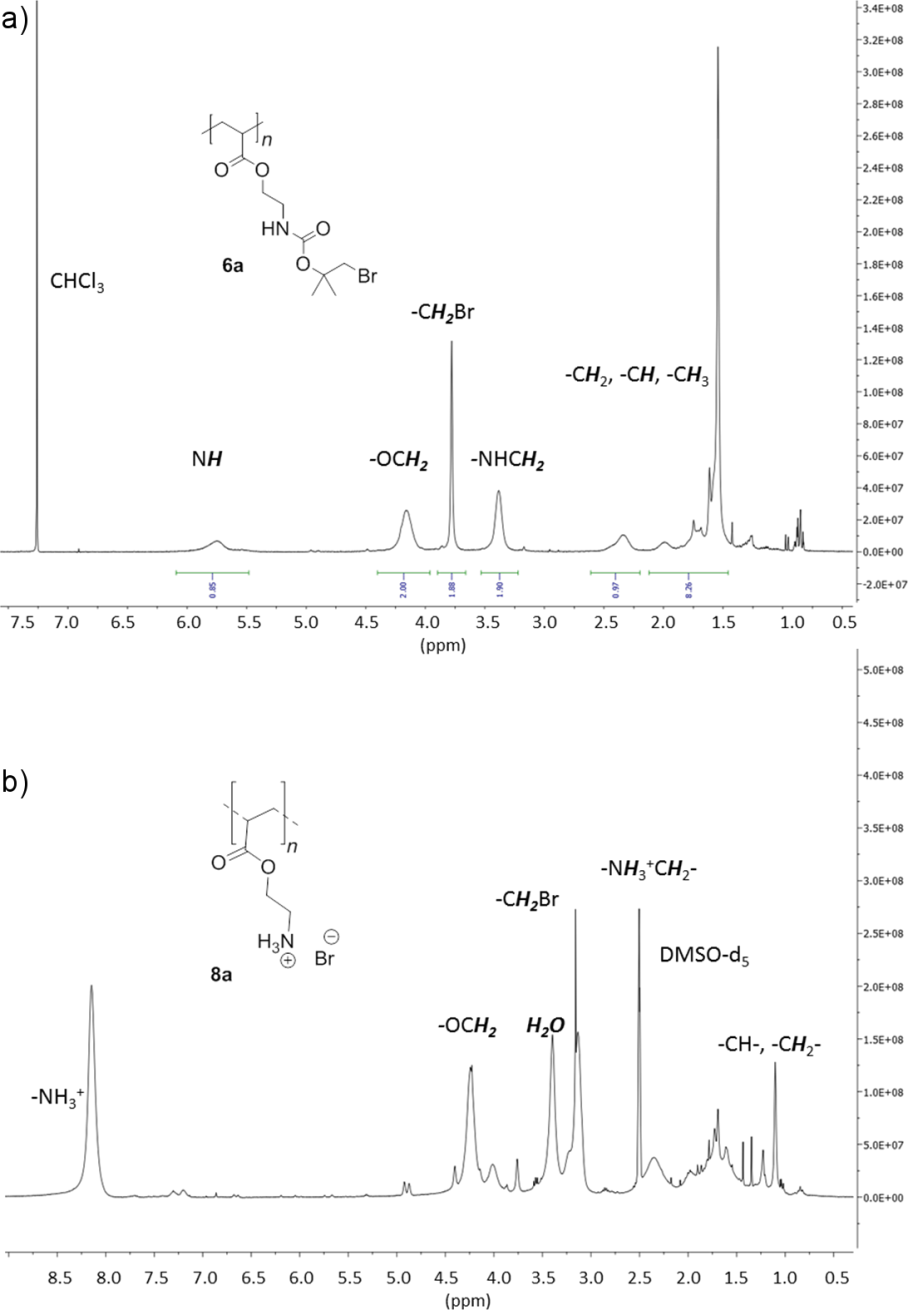
300 MHz ^1^H NMR spectra of a) **6a** in CDCl_3_ and b) **8a** in DMSO-*d*_6_.

The kinetic studies of **3a**, **6a** and **7a** are shown in Figure 4. Additionally, Figure 5 shows the ^1^H NMR spectra of homopolymer **6a** and deprotected polymer **8a**.

The calculated *t*_0.5_ and *t*_0.3_ values of deprotection for **3a**,**b**, **6a**,**b** and **7a**,**b** are summarized in Table 1.

**Table 1:** Half-times (*t*_0.5_) and *t*_0.3_ of deprotection for **3a**,**b**, **6a**,**b** and **7a**,**b**.

Compound	*t*_0.3_ [min]	*t*_0.5_ [min]

**3a**	84	180
**3b**	102	180
**6a**	162	276
**6b**	159	264
**7a**	114	192
**7b**	120	204

As assumed above the molecular dispersed monomer shows the highest reactivity followed by the copolymer. The solvolysis of the protecting group in the homopolymer is relatively slow because of neighboring group formed retarding hydrogen bonds (Scheme 4).

## Conclusion

It can be concluded from the above described results that the bromo-*tert*-butyloxycarbonyl group has a certain potential for the preparation of amino group containing functional (meth)acryl polymers. Since the deprotection takes place under mild conditions in polar solvents, some sensitive components, e.g., drugs, proteins, DNA may be present without being affected.

## Experimental

### Methods

^1^H NMR measurements were performed using a Bruker Avance 300 operating at 300 MHz at room temperature. Fourier transformation infrared spectroscopy (FTIR) was performed on a Nicolet 6700 FTIR spectrometer equipped with a diamond single bounce ATR accessory. The measurements were performed in the range of 400–3000 cm^−1^ at room temperature. Differential scanning calorimetry (DSC) was performed using a Mettler Toledo DSC 822 instrument equipped with a sample robot TSO801RO. The Apparatus was controlled over a temperature range between −50 °C and 350 °C at a heating rate of 15 °C min^−1^. The *T*_g_ values are reported as the average of five measurements using the midpoint method. The THF–GPC System compromises a Scharmbeck SFD degasser (Gastor BG12), a FLOW pump (Intelligent PUMP AL-12) and a Scharmbeck SFD (Model S5200) sampler. A Waters 486 Turnable Absorbance Detector and a Scharmbeck SFD RI 2000 detector were used for detection. A set of columns packed with porous styrene–divinylbenzene-copolymer beads was used for separation of the analytes (MZ Analysentechnik GmbH, 1 × guard column 100 Å, 3 × columns with 10,000, 1,000 und 100 Å). The system was calibrated with polystyrene standards with a molecular range from 575 g/mol to 3,114,000 g/mol. THF was used as eluent at a flow rate of 1 mL min^−1^.

### Materials

Commercial reagents and solvents were purchased from Sigma-Aldrich, Merck and Fluka. If not stated otherwise all chemicals were of analytical grade and were used as received without any further purification. 2-Isocyanatoethyl acrylate and (meth)acrylate were purchased from Shōwa Denkō K.K.

Chloroform-*d* (99.8 atom % D) was obtained from Deutero GmbH (Germany). All solvents were dried by standard methods. Column chromatography was performed using Acros Organics silica gel 60 (230–400 mesh) and thin layer chromatography (TLC) of the products using Merck silica 60 F254 plates.

### 1-Bromo-2-methylpropan-2-ol (**2a**)

In a 500 mL three-necked flask with a reflux condenser *tert*-butanol (100 g, 1.35 mol) was heated under reflux. A second 500 mL single-necked flask was equipped with *N*-bromosuccinimide (NBS, 50 g 0.28 mol) in a mixture of THF/H_2_O 1:2. To the boiling *tert*-butanol 5 portions of 5 mL each concentrated sulfuric acid were added dropwise every 15 min. The forming isobutene was bubbled into the NBS solution under vigorous stirring. After 45 min, all of the NBS disappeared and stirring was continued for an additional hour. THF was evaporated and the remaining aqueous mixture was extracted several times with diethyl ether. The extract was dried over magnesium sulfate and the solution was evaporated. The remaining oil was distilled to give **2a** as a pure colorless liquid: yield 22 g (51%); bp 55 °C at 25 mbar; ^1^H NMR (300 MHz, CDCl_3_) δ [ppm] 3.39 (s, 2H, -C*H*_2_-Br); 2.43 (s, 1H, -O*H*); 1.30 (s, 6H, -C*H*_3_); IR (diamond) 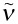 [cm^−1^]: 3380 (*s*, ν_OH_), 2976 (*m*, ν_C-H_), 2931 (*m*, ν_C-H_), 2872 (*m*, ν_C-H_), 1465 (*m*, ν_-CH2-)_, 1379 (*s*, ν_OH_).

### 1-Bromo-2-phenylpropan-2-ol (**2b**)

Into a stirring solution of *N*-bromosuccinimide (NBS, 32 g, 0.18 mol) in ^1^/_3_ THF and ^2^/_3_ H_2_O, α-methylstyrene (10 mL, 0.15 mol) were added. After 2 h of vigorous stirring, THF was evaporated and the aqueous solution was extracted several times with diethyl ether. The extract was dried over magnesium sulfate and the solution was evaporated. The crude product was purified by column chromatography using *n*-hexane/ethyl acetate 1:1 to give the desired colorless liquid **2b**: yield 21.35 g (66%). ^1^H NMR (300 MHz, CDCl_3_) δ [ppm] 7.57–7.54 (m, 2H, Ar*H*), 7.38–7.32 (m, 2H, Ar*H*), 7.27–7.22 (m, 1H, Ar*H*), 5.40 (s, 1H, -O*H*), 3.78 (s, 2H, -C*H*_2_-Br), 1.66 (s, 3H, -C*H*_3_); IR (diamond) 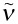 [cm^−1^]: 3442 (*s*, ν_OH_), 3027 (*m*, ν_C-H_), 2978 (*m*, ν_C-H_), 1602 (*w*, ν_C=C_), 1493 (*w*, ν_C=C_), 1446 (*m*, ν_-CH2-_), 1374 (*s*, ν_OH _*_tert_*_-alcohol_).

### 1-Bromo-2-methylbut-3-en-2-ol (**2c**)

Into a stirring solution of *N*-bromosuccinimide (NBS, 20 g, 0.12 mol) in ^1^/_3_ THF and ^2^/_3_ H_2_O, isoprene was added in 3 small portions of 5 mL (0.05 mol) at 0 °C. Stirring was continued for an additional hour. THF was evaporated and the remaining aqueous mixture was extracted several times with diethyl ether. The extract was dried over magnesium sulfate and the solution was evaporated. The remaining oil was distilled to give **2c** as a pure colorless liquid: yield 7.41 g (37.5%); bp 62 °C at 20 mbar. ^1^H NMR (300 MHz, CDCl_3_) δ [ppm] 5.88 (dd, 1H, -CH=C*H*H), 5.34–5.16 (m, 2H, -C**H**=C*H*_2_), 3.44 (s, 2H, -C*H*_2_-Br), 2.32 (s, 1H, -O*H*), 1.40 (s, 3H, -C*H*_3_); IR (diamond) 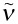 [cm^−1^]: 3412 (*s*, ν_OH_), 2980 (*m*, ν_C-H_), 2928 (*m*, ν_C-H_), 1644 (*m*, ν_C=C_), 1453 (*m*, ν_-CH2-_), 1371 (*s*, ν_OH_).

### General information for the preparation of the monomers

All syntheses were carried out under an argon atmosphere at room temperature using dibutyltin dilaurate (DBTL) as catalyst.

### 2-((1-Bromo-2-methylpropan-2-yl)oxycarbonylamino)ethyl acrylate (**3a**)

To a stirring solution of 2-isocyanatoethyl acrylate (**1a**, 8 g, 0.056 mol) and 2 mol % DBTL in 25 mL toluene, 1-bromo-2-methylpropan-2-ol (**2a**, 7.7 g, 0.05 mol) was added. After 24 h the solvent was evaporated and the crude product was purified by column chromatography using *n*-hexane/ethyl acetate 1:1 to give the pure product **3a**: yield 5.39 g (32%). ^1^H NMR (300 MHz, CDCl_3_) δ [ppm] 6.32 (dd, ^3^*J* = 17.3 Hz, ^2^*J* = 1.5 Hz, 1H, -CH=C*H*H), 6.04 (dd, ^3^*J* = 17.3 Hz, ^3^*J* = 10.4 Hz, 1H, -C*H*=CH_2_), 5.76 (dd, ^3^*J* = 10.4 Hz, ^2^*J* = 1.5 Hz, 1H, -CH=C*H*H), 5.22 (s, 1H, -N*H*-), 4.13 (t, ^3^*J* = 5.4 Hz, 2H, -OC*H*_2_), 3.67 (s, 2H, -C*H*_2_-Br), 3.34 (q, ^3^*J* = 5.6 Hz, 2H, -NH-C*H*_2_), 1.44 (s, 6H, -C*H*_3_); IR (diamond) 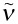 [cm^−1^]: 3358 (*m*, ν_N-H_), 2982 (*m*, ν_C-H_), 2936 (*m*, ν_C-H_), 1708 (*w*, ν_C=O_), 1635 (*w*, ν_C=C_), 1618 (*w*, ν_C=Ct_), 1516 (*s*, ν_N-H_), 983 (*s*, ν_CH=CH2_), 809 (*s*, ν_CH=CH2_).

### 2-((1-Bromo-2-methylpropan-2-yl)oxycarbonylamino)ethyl methacrylate (**3b**)

To a stirring solution of 2-isocyanatoethyl methacrylate (**1b**, 6 g, 0.039 mol) and 2 mol % DBTL in 25 mL toluene, 1-bromo-2-methylpropan-2-ol (**2a**, 5.8 g, 0.038 mol) was added. After 24 h the solvent was evaporated and the crude product was purified by column chromatography using *n*-hexane/ethyl acetate 1:1 to give the pure product **3b**: yield 5.3 g (43%). ^1^H NMR (300 MHz, CDCl_3_) δ [ppm] 6.12–6.11 (m, 1H, -C(CH_3_)-C*H*H), 5.59–5.57 (m, 1H, -C(CH_3_)=CH*H*), 4.95 (s, 1H, -N*H*-), 4.21 (t, ^3^*J* = 5.4 Hz, 2H, -OC*H*_2_), 3.75 (s, 2H,-C*H*_2_-Br), 3.44 (m, 2H, -NH-C*H*_2_-), 1.93 (s, 3H, -C*H*_3_), 1.53 (s, 6H, -C*H*_3_); IR (diamond) 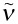 [cm^−1^]: 3365 (*m*, ν_N-H_), 2978 (*m*, ν_C-H_), 2929 (*m*, ν_C-H_), 1708 (*w*, ν_C=O_), 1637 (*w*, ν_C=C_), 1520 (*s*, ν_N-H_), 943 (*s*, ν_CH=CH2_), 814 (*s*, ν_CH=CH2_).

### 2-((1-Bromo-2-methylbut-3-en-2-yl)oxycarbonylamino)ethyl acrylate (**3d**)

To a stirring solution of 2-isocyanatoethyl acrylate (**1a**, 4.27 g, 0.03 mol) and 2 mol % DBTL in 25 mL toluene, 1-bromo-2-methylbut-3-en-2-ol (**2c**, 5 g, 0.03 mol) was added. After 24 h the solvent was evaporated and the crude product was purified by column chromatography using *n*-hexane/ethyl acetate 1:1 to give the pure product **3d**: yield 1.7 g (17%). ^1^H NMR (300 MHz, CDCl_3_) δ [ppm] 6.43 (dd, ^3^*J* = 17,3 Hz, ^2^*J* = 1.5 Hz, 1H, -CH-C*H*H ), 6.12 (dd, ^3^*J* = 17.3 Hz, ^2^*J* = 10.4 Hz, 2H, -C*H*-CH_2_), 5.86 (dd, ^3^*J* = 10.4 Hz, ^2^*J* = 1.5 Hz, 1H, -CH-C*H*H), 5.81–5.74 (m, 1H, -C*H*=CH_2_), 5.06 (s, 1H, -N*H*-), 4.58–4.46 (m, 2H, -CH=C*H*_2_), 4.24 (t, 2H, -OC*H*_2_), 4.01–3.98 (m, 2H, -C*H*_2_-Br), 3.50 (q, 2H, -NH-C*H*_2_), 1.73 (s, 3H, -C*H*_3_); IR (diamond) 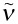 [cm^−1^]: 3348 (*m*, ν_N-H_), 2951 (*m*, ν_C-H_), 2869 (*m*, ν_C-H_), 1705 (*w*, ν_C=O_), 1636 (*w*, ν_C=C_), 1615 (*w*, ν_C=C_), 1528 (*s*, ν_N-H_), 983 (*s*, ν_CH=CH2_), 808 (*s*, ν_CH=CH2_).

### General information for the preparation of the polymers and copolymers

All polymerizations were carried out in toluene (75 wt %) at 50 °C using 5 mol % 2,2'-azobis(4-methoxy-2,4-dimethylvaleronitrile) (V-70) as initiator. After 24 h the polymerizations were stopped and the solutions were precipitated by pouring into *n*-hexane. The obtained polymers were filtered off and dried under vacuum.

### Poly-(2-((1-bromo-2-methylpropan-2-yl)oxycarbonylamino)ethyl acrylate) (**6a**)

To a mixture of **3a** (0.35 g, 1.19 mmol) in 0.7 g toluene, V-70 (5 mol %, 18.35 mg) dissolved in 0.35 g toluene was added. Polymer **6a** was obtained as a colorless solid: yield 0.28 g (80%). ^1^H NMR (300 MHz, CDCl_3_) δ [ppm] 6.04–5.45 (m, 1H), 4.33–3.97 (m, 2H), 3.90–3.60 (m, 2H), 3.55–3.24 (m, 2H), 2.53–2.12 (m, 1H), 2.09–1.66 (m, 2H), 1.61–1.54 (m, 6H); IR (diamond) 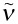 [cm^−1^]: 3359 (*m*, ν_N-H_), 2955 (*m*, ν_C-H_), 2930 (*m*, ν_C-H_), 1698 (*w*, ν_C=O_), 1516 (*s*, ν_N-H_); DSC: *T*_g_ = 93.1 °C; GPC (THF): 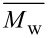 = 9300 g/mol, 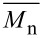 = 4400 g/mol, D = 2.11.

### Poly-(2-((1-bromo-2-methylpropan-2-yl)oxycarbonylamino)ethyl methacrylate) (**6b**)

To a mixture of **3b** (0.38 g, 1.23 mmol) in 0.76 g toluene, V-70 (5 mol %, 19.02 mg) dissolved in 0.38 g toluene was added. Polymer **6b** was obtained as a colorless solid: yield 0.34 g (89%). ^1^H NMR (300 MHz, CDCl_3_, rt) δ [ppm] 6.12–5.27 (m, 1H), 4.12–3.92 (m, 2H), 3.85–3.65 (m, 2H), 3.55–3.27 (m, 2H), 1.90–1.72 (m, 3H), 1.68–1.60 (m, 2H), 1.58–1.52 (m, 6H); IR (diamond) 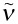 [cm^−1^]: 3353 (*m*, ν_N-H_), 2978 (*m*, ν_C-H_), 2934 (*m*, ν_C-H_), 1715 (*w*, ν_C=O_), 1521 (*s*, ν_N-H_), 1455 (*s*, ν_C-H (R-CH3)_); DSC: *T*_g_ = 125.9 °C; GPC (THF): 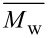 = 8900 g/mol, 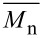 = 6000 g/mol, D = 1.48.

## References

[1] Burkhart A, Fischer J, Mondrzyk A, Ritter H (2014). Macromol Chem Phys.

[2] Mather B D, Viswanathan K, Miller K M, Long T E (2006). Prog Polym Sci.

[3] Thöne J, Ritter H (1987). Macromol Chem Phys.

[4] Rehse H, Ritter H (1989). Makromol Chem.

[5] Gormanns M, Rehse H, Ritter H (1991). Makromol Chem.

[6] Gormanns M, Ritter H (1993). Tetrahedron.

[7] Gormanns M, Ritter H (1994). Macromolecules.

[8] Carpino L A, Parameswaran K N, Kirkley R K, Spiewak J W, Schmitz E (1970). J Org Chem.

[9] Carpino L A (1973). Acc Chem Res.

[10] Carpino L A, Rice N W, Mansour E M E, Triolo S A (1984). J Org Chem.

[11] Ohnishi T, Sugano H, Miyoshi M (1972). Bull Chem Soc Jpn.

